# Exploring the application of machine learning to expert evaluation of research impact

**DOI:** 10.1371/journal.pone.0288469

**Published:** 2023-08-03

**Authors:** Kate Williams, Sandra Michalska, Eliel Cohen, Martin Szomszor, Jonathan Grant

**Affiliations:** 1 School of Social and Political Sciences, University of Melbourne, Melbourne, Victoria, Australia; 2 Policy Institute, King’s College London, London, Greater London, United Kingdom; 3 Electric Data Solutions, London, Greater London, United Kingdom; 4 Different Angles, Cambridge, Cambridgeshire, United Kingdom; Public Library of Science, UNITED KINGDOM

## Abstract

The objective of this study is to investigate the application of machine learning techniques to the large-scale human expert evaluation of the impact of academic research. Using publicly available impact case study data from the UK’s Research Excellence Framework (2014), we trained five machine learning models on a range of qualitative and quantitative features, including institution, discipline, narrative style (explicit and implicit), and bibliometric and policy indicators. Our work makes two key contributions. Based on the accuracy metric in predicting high- and low-scoring impact case studies, it shows that machine learning models are able to process information to make decisions that resemble those of expert evaluators. It also provides insights into the characteristics of impact case studies that would be favoured if a machine learning approach was applied for their automated assessment. The results of the experiments showed strong influence of institutional context, selected metrics of narrative style, as well as the uptake of research by policy and academic audiences. Overall, the study demonstrates promise for a shift from descriptive to predictive analysis, but suggests caution around the use of machine learning for the assessment of impact case studies.

## Introduction

While the term artificial intelligence (AI) often conjures up notions of entities with a general intelligence that resembles (and surpasses) human intelligence, much more common is the more modest and specific development and application of computational systems able to process information and make decisions that have traditionally required human intelligence. In the past decade, machine learning (ML)—one aspect of AI—has greatly advanced the capability of such systems, notably in visual detection of images [1, 2], language processing [3, 4], and speech and audio recognition [5, 6]. While major technical advances in capability are being driven mainly by big technology companies, some domains have begun exploring the practical implementation of these new technologies outside of technology-oriented environments. In particular, law [7], health [8], and cybersecurity [1] have begun to build capability towards using AI to improve accuracy and efficiency.

There is an increasing academic interest in more streamlined, less time-consuming alternatives to current peer review processes, which are used to determine the results of journal submissions, funding applications, and the outcomes of research quality assessments [9–11]. Prior research on peer review processes has identified often lengthy delays [12] as well as pervasive issues of bias [13–15] and superficiality [16]. Similarly, research has highlighted differences in the criteria used between disciplines [17] and between reviewers and journal editors [18]. Thus, a key question is whether emerging technologies can contribute to a leaner and fairer form of evaluation. Yet, the application of ML in research assessment is still rare, despite recent calls for improving current evaluation policy and practice [19]. At least in theory, the (semi)automated nature of ML can add to the overall objectivity, transparency and reproducibility of the process, potentially making it an attractive complement to peer review. However, there is limited published work that explores what this might look like in practice, particularly for large scale evaluation programs.

### Expert peer review in the UK’s Research Excellence Framework

The United Kingdom’s Research Excellence Framework (REF) is a national program that assesses the quality of research in UK higher education institutions. Its results determine the distribution of approximately £2 billion annual funding [20], as well as indirect outcomes that arise from improved reputation and increased student numbers. The UK has conducted research evaluations approximately every 5–7 years since 1986. Of these, REF2014 represented a substantial shift in the priorities and practices of the evaluation through the introduction of the explicit assessment of the wider societal and economic impact of academic research, which was absent from the previous iterations. The REF2014 submissions were evaluated along three main components—*outputs*, *impact* and *environment*—which accounted for 65%, 20% and 15% of the total score, respectively. Impact was assessed by ‘impact case studies’, which are structured narrative documents of four pages, designed to demonstrate the impact of a body of work conducted by academics within the submitting department. Impact was defined as “an effect on, change in or benefit to the economy, society, culture, public policy or services, health, the environment or quality of life, beyond academia” [21]. For REF2014, 154 institutions submitted 6,975 impact case studies, which were assessed by 36 sub-panels guided by four Main Panels [22].

Exceeding the previous rounds, REF2014 was estimated to cost almost £250 million [23], with the impact assessment component alone estimated at £55M [24]. Although the assessment was relatively cheap when compared to the amount spent on grant funding in the UK (estimated at 3% versus 13%) [24]), it nevertheless represents a sizeable financial burden for the sector. Despite the increasing cost of such a comprehensive and labour-intensive national evaluation, the latest round of assessment, REF2021, was conducted along largely similar lines of 2014 [10, 25]. As a specific mode of assessment, the UK’s system has been subject to criticism from some quarters while receiving support elsewhere. This occurs within the context of a broader body of literature on the challenges and flaws of the peer review process. Yet, despite the ongoing debates about the value of the exercise, one outcome of the REF has been the generation of a great deal of freely available information on the wider social and economic effects of the country’s publicly funded research [23].

### Emerging machine learning techniques for research evaluation

Researchers have begun to explore the potential of ML techniques in the area of research evaluation. One study by Yuan et al. [26] built a natural language processing (NLP) system to automate the peer review process, which they compared with expert evaluation. The results show that the NLP models were not as good as experts at overall summarising, but better at producing more comprehensive reviews. Researchers have also experimented with advanced multi-layered deep learning (DL) architectures for the prediction of ‘impactful’ research in medical science. DL is an engineering science based on multiple-layered artificial neurons propelled by large amounts of data and advances in computing power [27]. For instance, to explore impact, Nelson et al. [28] fed their DL model with publications’ metadata, title and abstract-level features to determine inclusion in patents, guidelines or policy documents, while Weis et al. [29] used paper, author, affiliation, journal and citation-based features in their DL model to predict its inclusion in the top 5% of the node centrality network after 5 years of publication. Both works favoured ML/DL approaches with many dimensions, which allow for sufficient expressivity and flexibility to capture complexity of established bibliographic *and* innovative semantic predictors of impact. One practical benefit of this approach is that DL is empirically driven, rather than guided by theory and prior assumptions. However, it is important to note that these types of technologies should not replace human judgment, but rather provide tools for producing evidence to support these judgements. If uncritically used, ML could have severe implications for research evaluation (for example, favouring the features of past successful cases at the expense of novel and unconventional cases).

Given the wealth of data arising from the evaluation, the existing literature on the REF2014 impact component provides some evidence of the potential for various ML tools to provide meaningful insights on large scale evaluations. An overview of the literature that analyses REF2014 impact submissions is summarised in Table 1. Much of this work primarily focuses on the qualitative data that arises from the universities’ narrative impact case studies. The approaches include topic modelling [25, 30], social network analysis [31, 32], and comparative linguistic analysis of high-scoring versus low-scoring submissions [33, 34]. These provide a wealth of information on the narrative features, such as the work of Reichard et al. [33], which used text mining to evidence statistically significant variation in writing style between the high-scoring and low-scoring impact case study submissions. In general, these analyses tend to focus mainly on particular disciplinary groupings or domains of interest, such as Business & Management [35] or Library & Information Studies [36], which limit their generalisability and potential for cross-domain insights. The review of the literature also shows that the application of more advanced ML techniques in research assessment is limited. That is, there is little work that uses these techniques in a predictive rather than descriptive manner. One notable exception is the work of Balbuena [9], who considered the quantitative metadata of REF2014 submissions. The study sought to replicate overall REF2014 Grade Point Average (GPA) results from data from Times Higher Education Report, the UK Higher Education Statistics Agency (HESA), the Web of Science, and the Guardian league tables using Bayesian Additive Regression Trees (BART) model [37]. A number of strong GPA predictors were identified, including how selective the university’s admissions standards were, the percentage of faculty with PhDs, and the average h-index of the faculty. The study concluded with a recommendation to complement peer review with ML in UK’s research assessment practice. Thus, so far, the use of advanced ML techniques has been limited to quantitative features. This highlights a key opportunity to combine the qualitative and quantitative aspects of the entire dataset using advanced ML techniques.

**Table 1:** Existing literature analysing REF2014 submissions.

Year	Study	Analysis	Dataset*
2021	‘Impact for whom? Mapping the users of public research with lexicon-based text mining’ [31]	Direct/indirect beneficiaries of research; Social Network Analysis (SNA).	3 AD
2021	‘Understanding the funding characteristics of research impact: A proof-of-concept study linking REF 2014 impact case studies with Researchfish grant agreements’ [38]	Relationship between research funding and research impact, characteristics of grant-linked ICS; descriptive statistics.	3 -
2020	‘Writing impact case studies: a comparative study of high-scoring and low-scoring case studies from REF2014’ [33]	Evidence of statistically significant differences in writing styles between high and low-scoring ICS across the Main Panels; text mining.	2 ABD
2019	‘Impact evaluation in Norway and in the UK: A comparative study based on REF 2014 and Humeval 2015–2017’ [34]	Comparison between the UK and Norwegian ICS (including linguistic aspect); semi-structured interviews.	-
2019	‘Achieving wider impact in business and management: analysing the case studies from REF 2014’ [35]	Engagement, utilisation and demonstration of impact demonstrated by discipline within Business & Management ICS; descriptive statistics.	1 -
**2018**	**‘The UK Research Excellence Framework and the Matthew effect: Insights from machine learning’** [9]	**Prediction of GPA from three types of variables: institutional, faculty and student characteristics; Bayesian Additive Regression Tree (BART).**	**2 -**
2018	‘How was social media cited in 2014 REF Impact Case Studies?’ [39]	Social media mentions across ICS, purpose of social media use in ICS; descriptive statistics.	3 -
2017	‘Evidencing Impact from Art Research: Analysis of Impact Case Studies from the REF 2014’ [40]	Search for the evidence-related codes (i.e. types of impact) within ICS; descriptive statistics.	1 ADE
2016	‘Impact case studies submitted to REF 2014: The hidden impact of nursing research’ [41]	Demonstrating an impact of nursing in other disciplines; descriptive statistics.	1 -
2016	‘Measuring the Impact of Research: Lessons from the UK’s Research Excellence Framework 2014 [30]’	Factors behind the impact scores (e.g. Q1 publications, number of researchers) demonstrated by UoA; Topic Modelling.	1 -
2016	‘Beyond Academia—Interrogating Research Impact in the Research Excellence Framework’ [42]	Impact interpretation (by Institution/by Panel); Topic Modelling—Descending Hierarchical Classification (DHC), Latent Dirichlet Allocation (LDA).	3 AD
2016	‘Beyond REF 2014: The impact of impact assessment on the future of information research’ [36]	Implications of impact assessment on future Library & Information Sciences researcher behaviour; semi-structured interviews with contributing academics.	1 -
2015	‘Digital Research Report: The Diversity of UK Research and Knowledge—Analyses from the REF impact case studies’ [32]	Demonstration of the interdisciplinary character of research in the UK; Social Network Analysis (SNA).	3 BDE

* 1–3 refers to the size of dataset (1 selected UoA, 2 selected domains, 3 entire REF2014 dataset,—not-specified), A-E refers to the relevant section of ICS used in analysis (A-Summary of the impact, B-Underpinning research, C-References to the research, D-Details of the impact, E-Sources to corroborate impact,—not-specified), and bold refers to the machine learning application for predictive analysis from REF2014.

By integrating qualitative and quantitative information from all REF2014 impact case studies, this article seeks to build upon this literature by exploring how ML techniques classify case studies that have been evaluated as high or low-scoring by expert panels. It provides an opportunity to consider the predictive potential (rather than descriptive potential) of such methods in relation to research assessment. The study addresses the following research questions:

**RQ1 -** Can we predict high-scoring impact case studies using ML?
**RQ2 -** What are the characteristics of high-scoring impact case studies?

## Methodology

The primary task was the binary classification of each individual impact case study as either high-scoring or low-scoring. The secondary task was the extraction of the specific features that were predictive of either high-scoring or low-scoring case studies. The following subsections specify the data sources used, the feature extraction process and the model development and interpretation stages.

### Data sources

#### REF submission data

6,637 REF2014 submissions (including impact scores) were downloaded from the official REF2014 website (https://ref.ac.uk/2014/). The impact case studies were structured in five sections: A) summary of the impact, B) underpinning research, C) references to the research, D) details of the impact, and E) sources to corroborate the impact. The case studies were scored by expert panels on a scale ranging from 4* (world-leading) down to 1* (recognised but modest), plus an extra ‘unclassified’ category. The published REF2014 impact scores were aggregated and scored at the submission level for each Institution and Unit of Assessment (UoA). The 154 submitting institutions made submissions in 36 UoAs that correspond to disciplinary groupings, so that submissions were primarily at a departmental level. Classifying the case studies as either ‘high’ or ‘low’ scoring for the purposes of this study was not immediately possible, given the scores of individual case studies were not published. To address this, following Balbuena [9], we distinguished between the highest and lowest-scoring submissions and assigned a GPA score to all studies in a given submission using the Eq 1, where 4*, 3*, etc. is the percentage of the relevant scores. There were 2 special cases in GPA estimation: 1) one case study associated with multiple institutions, and 2) one associated with multiple UoAs within the institution—in both cases we took the mean GPA for that case study.

$$\begin{array}{c} \text{IndividualGPAScore}=\frac{4\times 4^{*}+3\times 3^{*}+2\times 2^{*}+1\times 1^{*}}{100} \end{array}$$ (1)

To allow for further subsetting, we also collected other information that was used by REF2014 (https://impact.ref.ac.uk/casestudies/FAQ.aspx) to provide context to the submissions. Specifically, we recorded the overarching Main Panels that the UoAs fell under (S1 Table) and the institutions’ income categories from HESA (S2 Table), which had assigned universities to economic Peer Groups on the basis of income data in 2004–05.

Once a GPA was calculated for each case study, we ranked them in percentiles according to Main Panel and labelled 20% of the top case studies as high-scoring (1284) and 20% of the bottom case studies as low-scoring (1319). Normalisation by Main Panel (instead of top/bottom 20% on a global dataset) allowed a relatively equal distribution across departments for the extraction of more generic features. The distribution of case studies based on the estimated GPA scores across the panels (and the 20% top/bottom cut-off) is shown in S1 Fig. Based on the co-authors’ expertise in research evaluation, we opted for a 20% cut-off. This allowed investigation of the characteristics of very high and very low scoring case studies (whereas widening this threshold would make those characteristics less prominent).

#### Bibliometric data

Using the OpenAlex [43] API (api.openalex.org) we extracted the bibliometric data based on the DOIs (Digital Object Identifier) referenced in the case studies. To mirror the survey period used in REF2014, only citations from papers published in 2014 or earlier were considered. 23,967 DOIs were identified, and 21,263 were found in OpenAlex.

#### Policy citation data

The Overton database (https://www.overton.io/) was used for the extraction of policy citation data. Also in keeping with the REF survey period, a filter was applied to omit policy citations received after 2014. 3,959 of the 23,967 DOIs were cited more than once in Overton. A total of 16,992 citations came from the policy documents.

### Feature extraction

Using the whole dataset of case studies, we extracted six overarching groups of features: discipline, institution, explicit text, implicit text, bibliometric indicators and policy indicators. Each feature is set out in the context of its group, metric, and brief description in Table 2.

**Table 2:** Features extracted from REF2014 impact narratives.

Group	Feature	Metric	Description
Discipline	UoA	1 out of 36 units	Unit of Assessment
Institution	UKPRN	1 out of 198 institutions	UK Provider Reference Number
Explicit text	Narrative style	Maximum 5-grams; vocabulary size of 7,787	TF-IDF scheme applied to extract 7,787 terms with the highest weight followed by maximum 5-grams extraction; The distribution of n-gram sizes is 1: 6390, 2: 1317, 3: 64, 4: 12, 5: 4.
Implicit text	Readability^a^	Flesch Reading Ease	Formula using the number of syllables, the number of words, and the average sentence length [46]
		Smog Index	Formula using the number of words with 3 syllables or more and the number of sentences [50]
		Automated Readability Index	Formula using average sentence length and average word length [47]
		Dale Chall Readability Score	Formula using the number of difficult words not matching the Dale-Chall list of familiar words, number of words, and average sentence length [48]
		Difficult Words	Number of difficult words not matching the Dale-Chall list of familiar words [48]
	Sentiment^b^	10th, 25th, 50th, 75th, 90th	Percentile scores of sentiment values for each sentence in the text
Bibliometric indicators	Publication	DOI count	Count of DOIs referenced
		OA count	Count of DOIs with Open Access status
		Cites rank (mean)	Count of citations in full dataset
		Cites rank (max)	Count of citations in full dataset
	Author	UK author	Count of DOIs with one author in UK
		Non-UK author	Count of DOIs with at least one non-UK author
		Author-countries (mean)	Count of unique author-countries
		Author-countries (max)	Count of unique author-countries
	Author affiliation	Education	Count of institutions with the GRID^c^ type education
		Healthcare	Count of institutions with the GRID type healthcare
		Company	Count of institutions with the GRID type company
		Government	Count of institutions with the GRID type government
		Non-profit	Count of institutions with the GRID type non-profit
		Facility	Count of institutions with the GRID type facility
		Archive	Count of institutions with the GRID type archive
Policy indicators	Policy citations	Overton count	Count of DOIs that have at least one citation from policy
		Overton cites (max)	Count of Overton citations for any referenced DOIs
		Overton rank (max)	Count of Overton citations using category normalisation [51]

^a^ TextStat Python package;

^b^ TextBlob Python package;

^c^Global Research Identifier Database

The discipline feature group is concerned with the UoA of the submitting institution. This feature was constructed as a vector length of 36 (total number of units) where 1 represents the UoA responsible for submission while 0 represents the remaining 35 UoAs. The second group—institution—relates to the UK Provider Reference Number (UKPRN). This feature was constructed as a vector length of 198, where 1 represents the submitting institution.

For the impact case study text, we distinguished between implicit and explicit. The narrative style feature represents the explicit text included by the submitting institutions to demonstrate impact. We applied the TF-IDF (Term Frequency-Inverse Document Frequency) weighting scheme, which is often used in text summarisation and classification. TF-IDF allows extraction of the relevant and informative terms in the prediction task while excluding commonly occurring stop-words. It does this by assigning a higher weight to meaningful words and a lower weight assigned to common words. We also followed standard pre-processing steps, removing punctuation, diacritics, English stop words, terms that appear in either <3 or >50% of the case studies, URLs (keeping just domain), and converting to lower-case. We optimised for the ‘n-gram range’ (i.e. the number of the consecutive words used in feature set) and vocabulary size, which we detailed in S2 Fig. Here, we used all sections of the case studies (i.e. A-E) as the TF-IDF scheme naturally filters out words without meaningful contribution to the classification.

The readability and sentiment features represent the implicit aspects of case study text. The readability feature was given by five metrics that assign numerical scores to generate a ranking of books or other texts in order of ease of comprehension [44, 45]. These include the Flesch Reading Ease (FRE) metric ([46], the Automated Readability Index (ARI) [47], and Dale Chall Readability Score [48], which are now predominantly used in marketing, research and policy communication to measure the degree to which a piece of text is understood and engaged with [49]. For the sentiment feature, we calculated the percentiles of polarity scores in each sentence. Readability and sentiment were limited to sections A,B and D (as C and E include mainly references). This was to investigate whether there is an increased tendency to highlight the positive implications or results of research (e.g. case studies focusing on solutions as reflected in more positive sentiment), or rather highlight current gaps and limitations (e.g. case studies focusing on the problem as reflected in more negative sentiment).

The bibliometric indicators group contains characteristics derived from the publications that were included by submitting institutions to evidence their impact. This included three features: publication, which gives bibliometric counts; author, which classifies the location of authorship; and author affiliation, which provides categories of institution type from the Global Research Identifier Database (GRID) (https://www.grid.ac/). For this study, Open Access status included the labels ‘gold’, ‘bronze’, ‘green’, and ‘hybrid’. This set of features extends the work of Weis and Jacobson [29] by adding author affiliation metrics, designed to offer greater insight into inter-sector collaborations.

The final feature group is policy indicators. This represents the use of the submitted publications by policy contexts. Raw counts of policy citations are provided, as well as a category normalised metric to account for disciplinary differences.

### Model evaluation

The study used binary text classification (high-scoring/low-scoring) belonging to the supervised subcategory of ML and more broadly AI. Text classification is a “supervised learning task that is defined as the identification of categories of new documents based on the probability suggested by a specified training corpus of already labelled (identified) documents” [52]. The ML models used for such tasks range from well-established Support Vector Machines (SVM) and Random Forest (RF) to more recent Neural Networks (NN)-based classifiers. The conventional classifiers of SVM and RF were developed in the reverse order to the development of NNs. SVMs evolved from theory to implementation and experiments, while RF was based on idea that the performance of a set of many weak classifiers is usually better than a single classifier given the same quantity of trained information [53]. RF constructs many decision trees that are used to classify a new instance by the majority vote, and the suggested number of trees in a forest range between 64 and 128 [54] (we selected 100). NNs, on the other hand, followed a more heuristic path, from applications and extensive experimentation to theory [55].

Given the empirical nature of ML (and NN in particular), the comparison between the traditional approaches can be evaluated against NN as well as between the various architectures of NN themselves. For training the models, we used a proxy of a) the top 20% panel-normalised case studies to assign *the high-scoring label* and b) the bottom 20% panel-normalised to assign *the low-scoring label*. Training, in the context of ML, describes an automatic search process for better representations of the pre-specified classes/labels. We used two conventional classifiers (SVM and RF) and three NN-based architectures of increasing complexity. SVM and RF are the most straightforward, followed by net1, which has zero hidden layers, then net2, which has one hidden layer and 128 nodes, and finally, net3, which is an undercomplete autoencoder with four hidden layers and 250, 5, 5, 125 nodes [56]. For the SVM, we used the Linear Support Vector Classification. For all NN-based architecture, a ‘sigmoid’ activation function was used on the output. All hidden layers were densely connected and used the ‘relu’ activation function, except for net3 which uses ‘selu’, and were followed by a drop-out layer with a rate set to 0.3.

We conducted a comprehensive evaluation to: 1) compare the classification accuracy between models, 2) measure the relative contribution of different feature groups, 3) identify differences between the main panels and income categories. In total, we ran 5 × 23 × 12 experiments. This involved the five models (SVM, RF, net1, net2, net3), 23 feature group combinations (e.g. 1-explicit text & implicit text, 2-explicit text & implicit text & bibliometric indicators, etc.) and 12 (sub)sets (global dataset, four Main Panels, seven Income Categories). The application of multiple methods assures that variances are related to the features rather than the method [57].

The design used a stratified shuffle split, which involved randomly shuffling and dividing the dataset into ten parts, with nine parts used for training and one reserved for testing. This process was duplicated ten times, with each iteration reserving a different set for testing. This allowed us to measure the accuracy across the ten splits. The average accuracy was calculated for each model-feature-set scenario as explained above. The decision on which class each case study belongs to was based on the prediction score (PS), where *PS* = {0, 1} and a cut-off threshold was set at *PS* > 0.5 for class assignment. The evaluation was then based on the proportion of the correct predictions using the standard accuracy metric, where T-True, F-False, P-Positive, N-Negative (i.e. TP indicates case studies correctly identified as high-scoring, and TN indicates those correctly identified as low-scoring and so on). The standard accuracy metric uses the equation (Eq 2):

$$\begin{array}{c} \text{Accuracy}=\frac{\text{TP}+\text{TN}}{\text{TP}+\text{TN}+\text{FP}+\text{FN}} \end{array}$$ (2)

### Model interpretability

Prior work has confirmed that the performance of highly non-linear NN models—or ‘black box’ approaches—surpass the performance of the conventional classifiers such as SVM and RF (known as the performance versus explainability trade-off [58]). However, apart from simply providing a satisfactory classification system, the interpretability of the models’ internal workings is also a crucial element of our study. The overall idea behind NN is to determine which are the most useful representations of the input data in reflecting the expected output, in our case, the high-scoring/low-scoring case studies. This input-output transformation takes place in the hidden layers in the process of training on the labelled input data. The learning essentially occurs via finding the weights for all the nodes in the hidden layers that minimise the loss function (objective function) between the predictions and the targets. In the case of NN, the exact features that contribute towards the predictions are regarded as more opaque due to the highly non-linear and empirical character of the approach.

Although net1 and net2 have relatively basic architectures with zero and one hidden layers respectively, the main idea behind net3—the undercomplete autoencoder—is to learn a highly compressed input representation via the so called ‘bottleneck’, which is a hidden layer with a limited number of neurons. That is, the complete architecture in our case consists of four hidden layers with 250 neurons (1st), five neurons (2nd and 3rd—the compressed representations), and 125 neurons (4th). Autoencoders aim to desensitise the irrelevant features during encoding and compress the most meaningful ones during decoding while reconstructing an initial input (in our case, the impact case studies and the assigned label). Here, although it is difficult to elucidate precisely how a model works, autoencoders can nonetheless provide useful insights for practitioners and end users of ML [59]. The ML/DL approach can be used efficiently as a first pass assessment to identify areas for further exploration. This is a particularly useful approach when dealing with high-volume and high-variety contexts for which traditional methods can prove limited [60].

For the purposes of interpretability, the advantage of the traditional SVM and RF classifiers lies in their ease of extracting the predictive coefficients from the model. These techniques are highly popular and long-standing supervised ML algorithms used for both classification and regression problems, and detailed information can be found in [54, 61]. Thus, we followed standard procedure in extracting the SVM and RF coefficients to better understand which particular features contributed towards classification as high- or low-scoring (i.e. a direction indicative of the predicted class and a magnitude indicative of the relative importance in terms of SVM, and a magnitude indicative of the relative importance in terms of RF).

## Results & discussion

### Classification accuracy

This section considers how accurately the ML models classify high-scoring versus low-scoring REF impact submissions (RQ1). Here, we explore the models’ ability to predict GPA score from REF impact case studies given our set of six feature groups.

The experiments demonstrated the effects of the feature groups on the classification outcome. We compared the classification of our ML models on various combinations of groups to measure the predictive contribution of each. Across all models, there was a strong influence of institutional and disciplinary information (Table 3). Accordingly, we split the analysis into inclusive and exclusive of institution and discipline. By excluding these, the effect of the remaining feature groups became more prominent (Table 4). On average across the models, the classification ranged from 58.5% (just discipline) up to 90.4% (institution, discipline and policy indicators). After exclusion of institutional information, the maximum classification was 77.8% (the average across the models) for the explicit, implicit and bibliometric groups. Model training included explicit features, which were then filtered out when ranking important features. This finding indicates high fidelity of prediction from the qualitative submission narrative (both the words used directly and the latent metrics derived secondarily from the text) as well as quantitative information on publication, author and author affiliation.

**Table 3:** Accuracy across feature groups (incl. discipline & institution).

Category	svm	rf	net1	net2	net3	Mean
discipline	58.1%	60.1%	58.2%	57.4%	58.6%	**58.5%**
institution	82.5%	83.0%	83.3%	83.9%	83.0%	**83.1%**
discipline & institution	87.0%	99.3%	86.3%	98.7%	87.5%	**91.8%**
discipline & explicit text	79.1%	75.0%	78.0%	79.3%	78.7%	**78.0%**
discipline & implicit text	67.9%	64.4%	61.5%	66.1%	65.7%	**65.1%**
discipline & bibliometric indicators	67.7%	64.8%	65.7%	66.6%	65.4%	**66.1%**
discipline & policy indicators	63.0%	61.5%	61.6%	63.5%	62.8%	**62.5%**
discipline & institution & explicit text	89.2%	76.1%	87.7%	89.8%	88.2%	**86.2%**
discipline & institution & implicit text	87.2%	83.6%	86.1%	98.0%	87.4%	**88.5%**
discipline & institution & bibliometric indicators	88.1%	85.6%	86.3%	98.5%	86.5%	**89.0%**
discipline & institution & policy indicators	87.1%	94.3%	84.9%	98.5%	87.4%	**90.4%**
discipline & bib. & pol. & impl. & expl.	80.6%	76.2%	80.2%	81.4%	81.8%	**80.0%**
discipline & institution & bib. & expl. & impl.	89.7%	77.2%	88.1%	88.2%	88.4%	**86.3%**
discipline & institution & pol. & expl. & impl.	90.5%	76.0%	88.2%	89.2%	88.2%	**86.4%**
discipline & institution & bib. & pol. & expl. & impl.	88.6%	76.4%	87.9%	89.5%	88.8%	**86.2%**

**Table 4:** Accuracy across feature groups (excl. discipline & institution).

Category	svm	rf	net1	net2	net3	Mean
explicit text	74.2%	73.4%	77.0%	77.5%	75.2%	**75.5%**
implicit text	60.9%	59.1%	49.7%	57.8%	60.1%	**57.5%**
bibliometric indicators	60.3%	60.8%	56.7%	62.5%	62.5%	**60.6%**
policy indicators	57.5%	55.3%	52.8%	59.3%	59.2%	**56.8%**
explicit text & implicit text	77.7%	73.9%	77.7%	78.7%	76.9%	**77.0%**
explicit text & implicit text & bibliometric indicators	77.2%	75.4%	77.4%	78.7%	77.5%	**77.2%**
explicit text & implicit text & policy indicators	76.9%	75.6%	77.4%	78.1%	78.3%	**77.3%**
expl. & impl. & bib. & pol.	77.8%	75.6%	78.4%	77.9%	79.0%	**77.8%**

To explore these findings further, we trained separate models on subsets of the data representing each of the REF’s four Main Panels and the income category Peer Group of the submitting institutions. For each, we used the same combinations of feature categories as the global dataset above and also split the groups into inclusive and exclusive of discipline and institution information.

**Fig 1:**
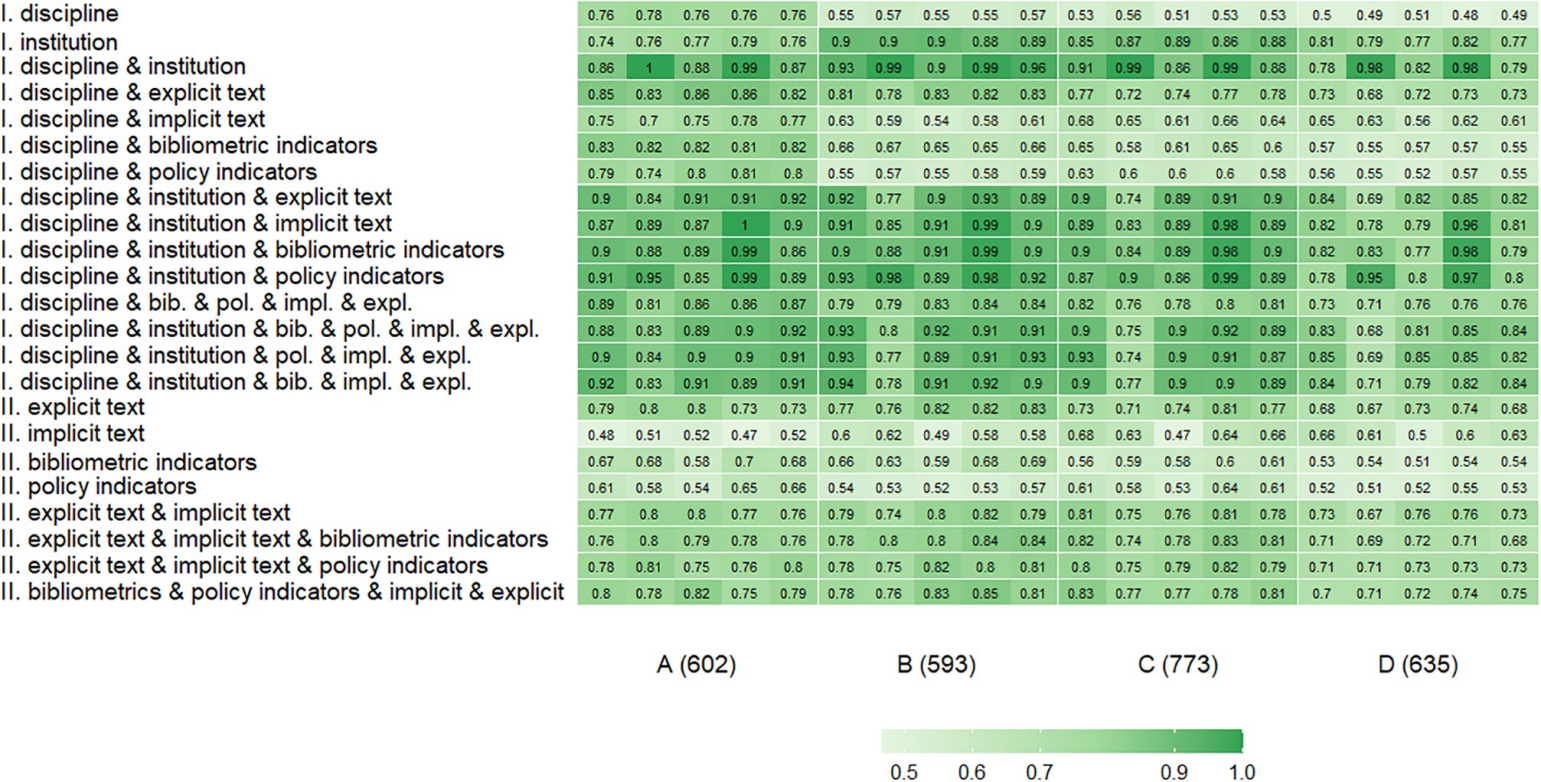
Classification accuracy by Main Panel: I. With institution and discipline, II. Without institution and discipline (column 1—svm, column 2—rf, column 3—net1, column 4—net2, column 5—net3).

In terms of the Main Panels, the comparative accuracy of the classification for each scenario is shown in Fig 1. Overall, predictions were stronger among Panels A (medicine, health and life sciences), B (physical sciences, engineering and mathematics) and C (social sciences) in comparison with Panel D (arts and humanities), particularly when institution and discipline were included. In Panels B, C and D, the implicit text features (readability, sentiment) were most predictive in comparison with Panel A. There was also a stronger impact of bibliometric and policy indicator groups in Panel A than in Panel D. The addition of more than three feature groups did not improve the classification accuracy on average.

**Fig 2:**
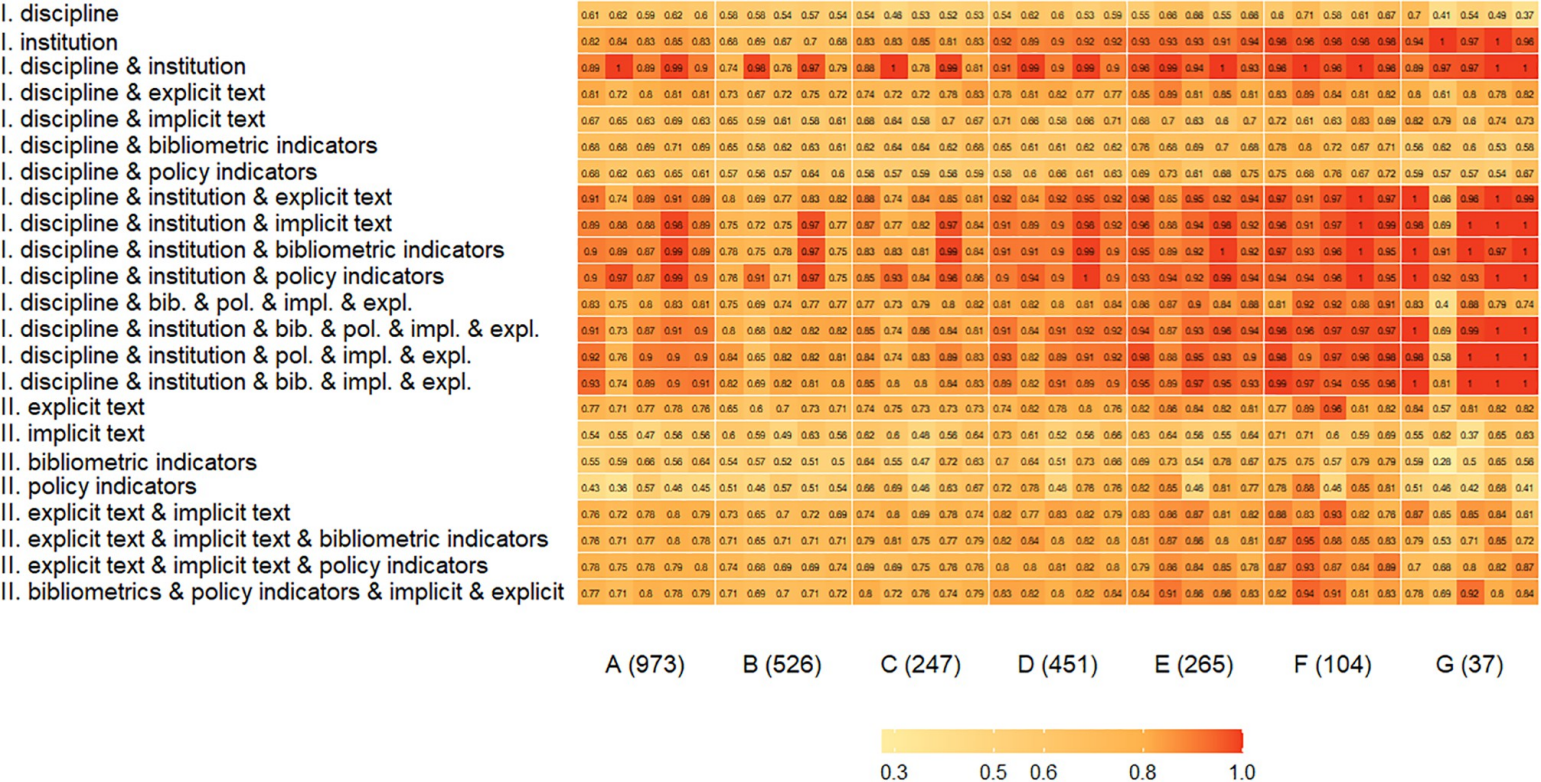
Classification accuracy by income category Peer Group: I. With institution and discipline, II. Without institution and discipline (column 1—svm, column 2—rf, column 3—net1, column 4—net2, column 5—net3).

For the income category Peer Groups, the comparative accuracy is shown in Fig 2. Greater predictive capacity was observed among the lower income groups D (research income of between 5% and 8% of total income), E (teaching institutions) and F (smaller teaching institutions) on average, compared to the higher income groups A (Russell Group, which are traditionally considered the most research-intensive and prestigious universities in the UK), B (research income of 22% or more of total income) and C (research income of between 8% and 21% of total income). As above, this was particularly true when institution and discipline were included. The models can more easily predict the high- or low-scoring case studies from the top Peer Group (A) or lower Peer Groups (D, E, F), whereas the middle income categories (B, C) are less clear. The exception was Peer Group G (specialised music/arts teaching institutions), due to a much smaller sample size (37). In addition, more than three feature groups did not improve accuracy. Future work would need a larger sample size to validate this, especially for Peer Groups C, E and F.

Previous analyses of REF impact results have identified that the assessment process seems to privilege certain disciplines and, typically, more prestigious, better-resourced universities [62, 63]. The novel contribution that our analysis makes to this evidence is an estimate (or more specifically, a number of estimates) of the extent to which discipline and institution predict the outcomes of REF impact assessments. As an illustration, when extracting the most important features for the classification of high scoring case studies (including institutional information), between 60% (svm model) and 77% (rf model) of the top 30 institutions belonged to the Russell Group.

### Predictive features

This section considers the insights that can be gained from ML models in terms of the characteristics of high-scoring REF submissions (RQ2).

We narrowed this section of the analysis down to the following feature groups: implicit text (readability, sentiment), bibliometric indicators (publication, author, author affiliation) and policy indicators (policy citations). Here, the explicit text feature group was excluded because of the amount and format of the data (i.e. around 7,787 n-grams, which would obscure the other findings). Given the prevalence of institutions’ names and locations which would reveal specific institutions, we did not include the top K words by feature weights in the analysis. To affirm the findings derived from standard procedures around the use of SVM coefficients, the feature weights were taken for both SVM and RF and each of the 10 folds (final average was taken). The weights were then ranked and the average of those ranks for both models (final rank) is shown in Table 5.

**Table 5:** Features important for high scoring case studies classification (svm and rf models coefficients).

Rank	Group	Feature	svm	rf
1	implicit text	*Difficult Words (DW)*	1.8724	0.0064
2	bibliometric indicators	*Cites rank (mean)*	0.6307	0.0075
3	bibliometric indicators	*Cites rank (max)*	0.4360	0.0073
3	policy indicators	*Overton cites (max)*	1.1266	0.0024
5	implicit text	*Flesch Reading Ease (FRE)*	1.2194	0.0017
6	implicit text	*Automated Readability Index (ARI)*	1.0041	0.0009
7	policy indicators	*Overton count*	0.3728	0.0020
8	policy indicators	*Overton rank (max)*	0.0816	0.0033
9	bibliometric indicators	*Healthcare affiliation*	0.1344	0.0006
10	bibliometric indicators	*Author countries (max)*	0.2219	0.0004
11	implicit text	*Sentiment-75th percentile*	0.0126	0.0009
12	implicit text	*Sentiment-90th percentile*	0.0369	0.0008
13	implicit text	*Sentiment-50th percentile*	0.0564	0.0005
13	implicit text	*Sentiment-10th percentile*	0.0672	0.0005
15	bibliometric indicators	*Company affiliation*	0.4358	0.0001
16	bibliometric indicators	*UK author*	0.0687	0.0003
17	implicit text	*Dale Chall Readability Score (DCRS)*	-0.6002	0.0013
17	implicit text	*Smog Index (SI)*	-0.3819	0.0009
19	bibliometric indicators	*Archive affiliation*	0.1229	0.0000
20	bibliometric indicators	*Author countries (mean)*	-0.5477	0.0007
20	bibliometric indicators	*DOI count*	-0.1209	0.0004
22	bibliometric indicators	*Government affiliation*	0.0401	0.0001
23	bibliometric indicators	*Non-UK author*	-0.1113	0.0002
24	bibliometric indicators	*Education affiliation*	-0.3393	0.0004
25	bibliometric indicators	*Non-profit affiliation*	0.0024	0.0001
26	bibliometric indicators	*Facility affiliation*	-0.2102	0.0001
27	implicit text	*Sentiment-25th percentile*	-0.4717	0.0002
27	bibliometric indicators	*OA count*	0.0000	0.0000

Interestingly, particularly prominent across high-scoring case studies were the readability features. The average of FRE, SI, ARI, DCRS metrics for high-scoring case studies indicated a more straightforward writing style. The strong finding around writing style builds on the work of Rechaird et al. [33], which emphasised the influence of latent characteristics derived from the submitted impact narratives. Thus, as well as the significance, reach and attribution of impact, as given by the REF’s published criteria, the presentation of the narratives seems also to have influenced impact assessment.

Overall, policy indicators were strongly predictive of high-scoring case studies. The study provided more granular insight into the relevance of specific policy-related features. In order of relevance, these were: 1) the maximum number of Overton citations for referenced DOIs (e.g. 1 highly-cited paper in policy documents), 2) the number of DOIs that have at least one citation in policy documents, and 3) the maximum citation percentile using category normalisation as in the work of Szomszor [51]. This reinforces the finding that there is a link between expert evaluation of impact and policy citation rank [51], and highlights the importance of policy influence to wider notions of research impact. Still, Smith et al. [64] (and based on wider discussion in Boswell et al. [65]) caution against rewarding individual researchers for ‘achieving’ research impact based on narrow indicators, such as citations in policy documents.

Publication-related features extracted from the OpenAlex database were also highly predictive of the high-scoring submissions. In order of importance, these were: 1) the mean rank of citations and 2) the maximum rank of citations. In terms of the affiliated sectors (the count of institutional affiliations with the selected sector according to GRID type), the strongest association with high-scoring case studies was found for company affiliation, followed by the healthcare and government affiliations. On the other hand, those related to archive, non-profit, education and facility affiliations were more predictive in low-scoring cases, possibly indicating that assessors hold assumptions about the kinds of collaborators that are most valuable for impact. As for the countries of the authors, ‘the number of DOIs with at least one non-UK author’ feature was more predictive of low-scoring examples (although the strength of the association was relatively small). Sentiment or open access (OA)-level features (count of DOIs with OA status) were not strongly predictive of either high or low-scoring classifications.

## Conclusions

We ran a series of experiments to investigate the ability of ML techniques to predict and elucidate the results of a large-scale expert evaluation system. By training five models on a range of qualitative and quantitative feature groups of REF2014 impact submissions, we showed that ML techniques are able to predict high-scoring and low-scoring case studies. We then used trained model weights to identify the key characteristics of high-scoring submissions.

The approach we used is novel because of the inclusion of both quantitative and qualitative feature groups (including both explicit and implicit qualitative features). Previous literature has focused on the selection and evaluation of *either* qualitative or quantitative features, such as the narrative components of the assessment [33] or the external metadata [9]. Our approach thus sought to build on this literature by further drawing out the complex holistic nature of the expert evaluation. It represents a step towards a *predictive* approach to the analysis of REF impact case studies, in contrast with the currently dominant *descriptive* approaches (in line with big data analytics [66]).

The results provide insight into the characteristics of impact submissions that mattered in REF2014. As in the global dataset, discipline and institution were shown to be strongly predictive of high scores when the models were trained on the Main Panel and Peer Group subsets. This supports the notion that the rules and traditions within research fields shape what counts as meaningful impact, and that some disciplines may find it easier to evidence more recognisable forms of impact. It also suggests that the evaluation captures something about the universities’ material resources or prestige. Thus, it may be that REF2014 was not entirely able to avoid the social conditions that surround specific institutions. That is, there may have been implicit pre-conceptions that were captured in the evaluation.

We chose to focus this analysis on the REF2014 evaluation in order to compare results to existing work on the same publicly available data. We sought to explore the potential of this line of inquiry and develop a proof-of-concept approach. Particularly as the number of studies on REF2021 grow, future research could fruitfully apply this framework to the most recent REF data. Key limitations of this study include that the assumed ‘gold standard’ of high/low-scoring labels were only estimates, given the that the exact scores at the individual case study-level were not made publicly available. In addition, the findings directly reflect the REF2014 data, and for wider generalisation (in terms of both accuracy and predictive features) more data is needed. For example, this may take the form of data from several REF iterations or integrating similar case studies from outside of the UK. This is especially true for NN architectures that learn from the examples rather than rules set a priori.

This study illuminates the key question of whether ML techniques can contribute to a leaner and fairer form of evaluation. Our analysis suggests that while these techniques may offer the potential for leaner evaluation, a high degree of caution is required around expecting ‘fairness’. This is because of the influence of past characteristics of high-scoring case studies at the expense of emerging and infrequent ones. Using the wealth of data provided by REF2014, we have demonstrated how an ML approach to evaluation might have looked in practice and what characteristics may have been emphasised and with what accuracy.

Overall, our work makes two key contributions. First, it shows that ML models are able to accurately classify case studies that have been evaluated as high or low-scoring by expert panels. It thus demonstrates that ML architectures are able to process information to make decisions that resemble the conclusions of expert evaluators. In doing so, it highlights the possible role of emerging technologies that can learn, and help us learn, about the wealth of valuable data generated by assessments such as the REF. Second, it demonstrates the value of ML in illuminating the features of case studies that seem to influence peer reviewers’ assessments. The features extraction elicited the types of characteristics that may be favoured if an ML approach was applied for their automated assessment. Most significantly, this included the institution and disciplinary context in which the researchers being assessed are embedded, but also strongly highlighted the way that the narratives are written, the uptake by policy and academic audiences, and the sector affiliation of collaborators.

## Supporting information

S1 Table REF2014 Units of Assessment (UoA) and overarching Main Panels. The Main Panels can be broadly grouped as follows: A is medicine, health and life sciences, B is physical sciences, engineering and mathematics, C is social sciences and D is arts and humanities.
(TEX)

S2 Table REF2014 UKPRN and institutional Peer Groups based on HESA income categories for 2020–21. References to income are to 2012–13 HESA data, where Peer group A: Institutions with a medical school and research income* of 20% or more of total income; Peer group B: All other institutions with research income* of 15% or more of total income; Peer group C: Institutions with a research income* of between 5% and 15% of total income; Peer group D: Institutions with a research income* less than 5% of total income and total income greater than £150M; Peer group E: Institutions with a research income* less than 5% of total income and total income less than or equal to £150M; Peer group F: Specialist music/arts teaching institutions (*research income is defined as the funding council recurrent research grant plus the total research grants and contracts returned in the 2012–13 HESA Finance Statistics Return (FSR)).
(TEX)

S1 Fig Distribution of calculated GPA scores of impact case studies by Main Panels. (TIFF)

S2 Fig Optimal vocabulary size and ngram range that returns maximum accuracy (using tf-idf scheme). (TIFF)
