# Supplementary figures and images for: Exploring the application of machine learning to expert evaluation of research impact
Source: PLoS One. 2023 Aug 3;18(8):e0288469. doi: 10.1371/journal.pone.0288469 (PMC10399885; doi:10.1371/journal.pone.0288469)

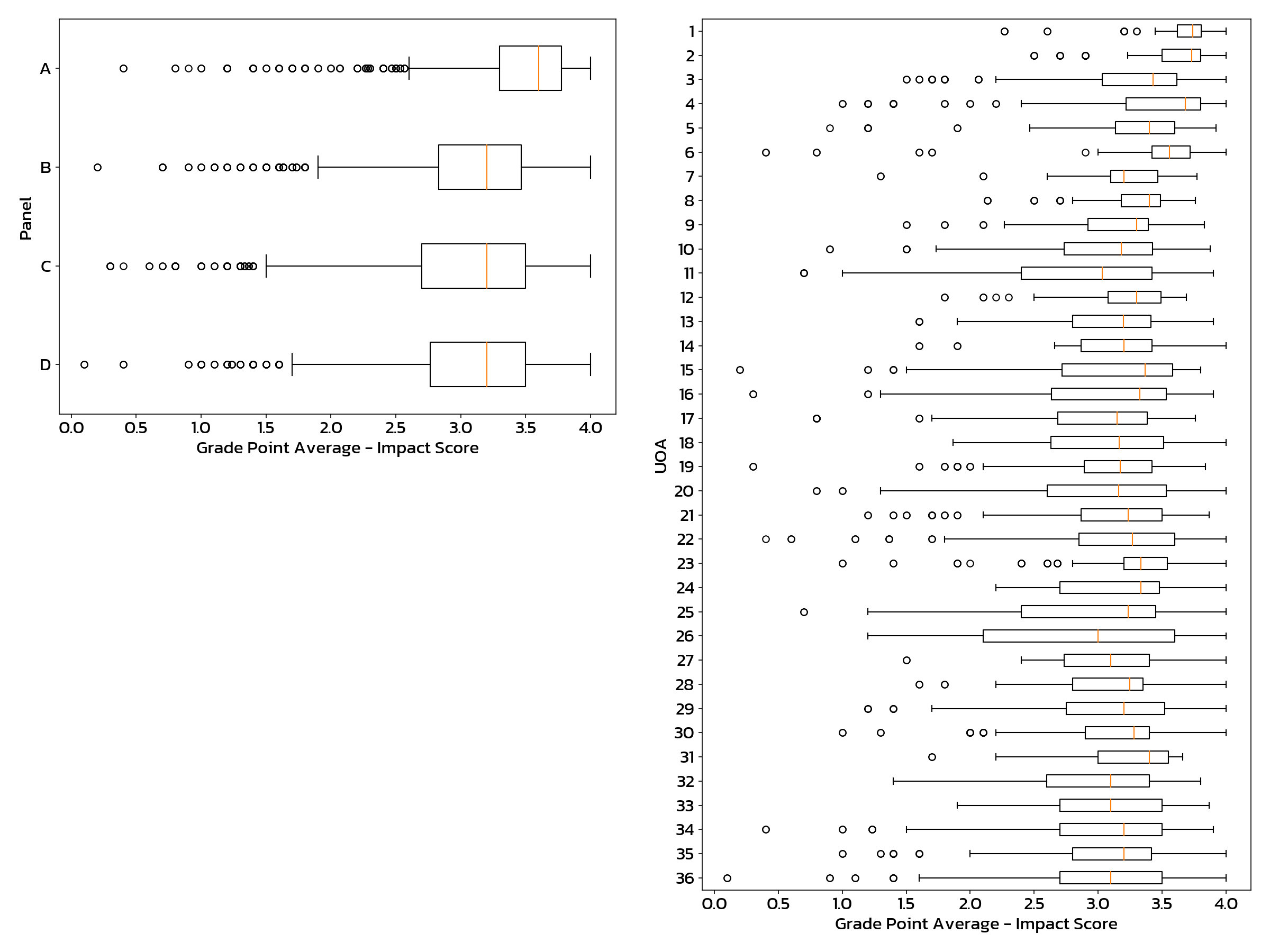

Supplement: S1 Fig — (TIFF) [file pone.0288469.s003.tiff]

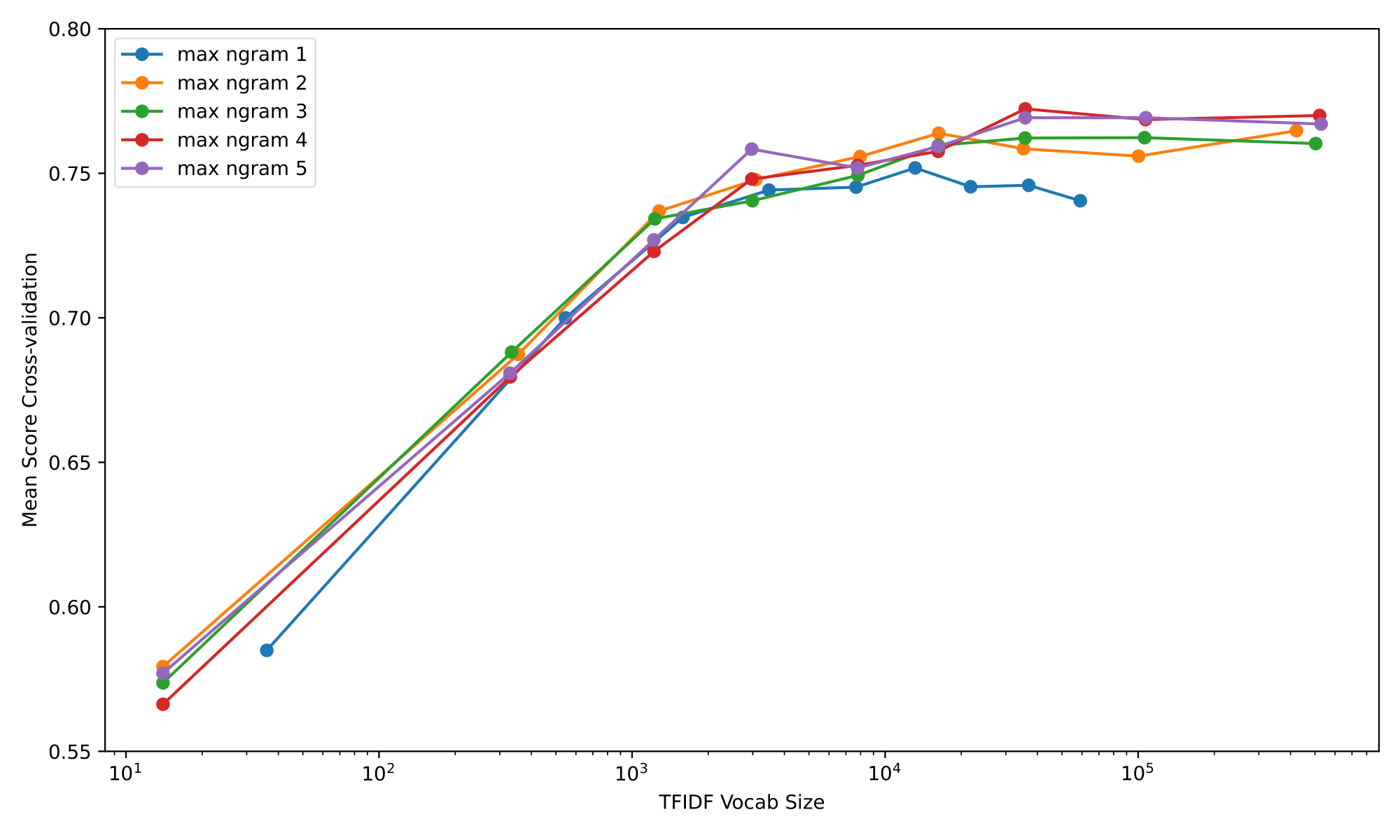

Supplement: S2 Fig — (TIFF) [file pone.0288469.s004.tiff]
